# Enhanced Reactivity of [Hydroxy(tosyloxy)iodo]benzene in Fluoroalcohol Media. Efficient Direct Synthesis of Thienyl(aryl)iodonium Salts

**DOI:** 10.3390/molecules15031918

**Published:** 2010-03-17

**Authors:** Motoki Ito, Chieko Ogawa, Nobutaka Yamaoka, Hiromichi Fujioka, Toshifumi Dohi, Yasuyuki Kita

**Affiliations:** 1 Graduate School of Pharmaceutical Sciences, Osaka University, 1-6 Yamada-oka, Suita, Osaka, Japan; 2 College of Pharmaceutical Sciences, Ritsumeikan University, 1-1-1 Nojihigashi, Kusatsu, Shiga, Japan

**Keywords:** [hydroxyl(tosyloxy)iodo]benzene, fluoroalcohol solvents, diaryliodonium salts, thiophenes, single-electron-transfer

## Abstract

In this manuscript, we report clear evidence for the generation of aromatic cation radicals produced by using [hydroxy(tosyloxy)iodo]benzene (HTIB) in fluoroalcohol solvents such as 2,2,2-trifluoroethanol (TFE) and 1,1,1,3,3,3-hexafluoro-2-propanol (HFIP). The single-electron-transfer (SET) oxidation ability of HTIB to give cation radicals was first established by ESR and UV measurements. The reaction was broadly applied to various thiophenes, and unique thienyliodonium salts were directly synthesized by this method in excellent yields without the production of any harmful byproducts.

## 1. Introduction

In the last quarter of a century, the chemistry of hypervalent iodine has been extensively studied due to the increasing importance of greener synthetic processes [1,2,3,4,5,6,7,8,9]. A number of iodine(III) and iodine(V) reagents, e.g., phenyliodine(III) diacetate (PIDA), phenyliodine(III) bis(trifluoroacetate) (PIFA), [hydroxyl(tosyloxy)iodo]benzene (HTIB, Koser’s reagent), iodosobenzene, Dess-Martin periodinane (DMP), and 2-iodoxybenzoic acid (IBX), have become commercially available and are routinely used in modern organic synthesis due to their useful oxidizing properties, high stability, and low toxicity, by which they are recognized as environmentally benign alternatives to highly toxic heavy metal oxidants including lead (IV), mercury (II), cadmium (IV) and thallium (III) elements. 

**Scheme 1:**
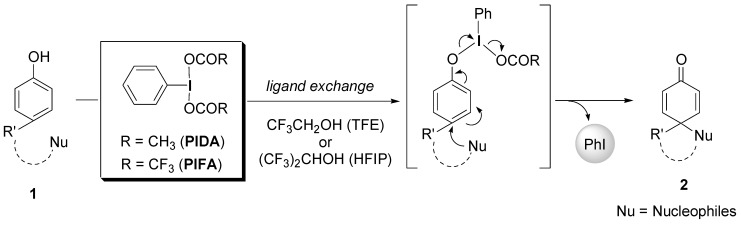
Phenolic oxidations using hypervalent iodine(III) reagents.

In our continuing study in the field of hypervalent iodine chemistry [10,11,12,13,14,15,16,17,18,19,20,21,22,23,24], we have successfully introduced highly polar, but low nucleophilic fluoroalcohol solvents, that is, CF_3_CH_2_OH or (CF_3_)_2_CHOH, for the first time to the hypervalent iodine-mediated oxidative transformation as stabilizing solvents of the reactive cationic intermediates [25,26,27,28]. The oxidations of phenols **1** using PIDA or PIFA should take place via two-electron transfer processes that involve the initial ligand exchange of the phenolic oxygens at the iodine centers (Scheme 1). During the successive reductive elimination step, various functionalized cyclohexadienones **2** would be produced by attack of the nucleophiles at the *para*- or *ortho*-positions of the phenol rings [10,11,12,13,14,15,16]. 

**Scheme 2:**
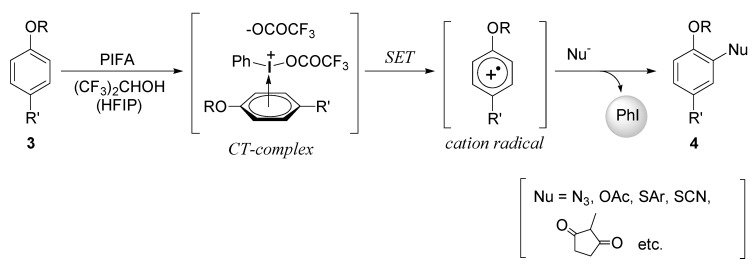
Generation of cation radical intermediates by alternative SET oxidations.

For the phenyl ethers, we also discovered the use of PIFA in fluoroalcohol solvents that permitted novel aromatic substitution by nucleophiles (Scheme 2) [17,18,19,20]. Usually, the phenylethers **3** are inert to the iodine(III) reagents in other typical solvents. We established the formation of aromatic cation radicals as the key intermediates of the reactions, which were generated by the single-electron-transfer (SET) oxidation through the charge-transfer (CT)-complex of phenyl ethers **3** and PIFA. This is the first report that the iodine(III) reagents showed a SET oxidation ability for the oxidations [18]. As the cation radical intermediates would be effectively stabilized by the fluoroalcohol solvents, the nature of the solvent was crucial for the success of the reactions, and the use of CH_2_Cl_2_ instead of the fluoroalcohol solvents diminished the yield of the aromatic substitution products **4**. 

**Figure 1:**
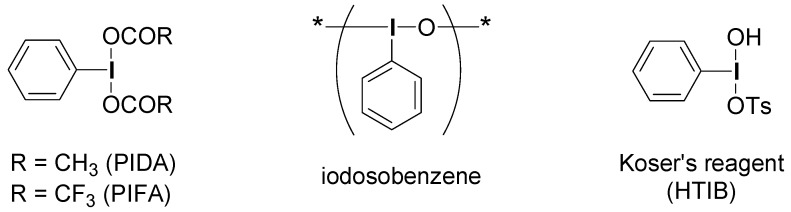
Representative hypervalent iodine(III) reagents.

Regarding the reagents for the SET oxidations, only a few iodine(III) reagents are known to date except for PIFA, PIDA, and iodosobenzene (Figure 1). Based on the analogy with PIFA and HTIB, Koser *et al.* proposed in 2006 a SET reaction mechanism for the nucleophilic substitution of certain polyaromatic hydrocarbons using HTIB in CH_2_Cl_2_ [29]. However, there was no clear evidence and spectroscopic determination of the reactive intermediates in the literature, and thus the SET oxidation ability of HTIB has not been elucidated until now.

**Scheme 3:**
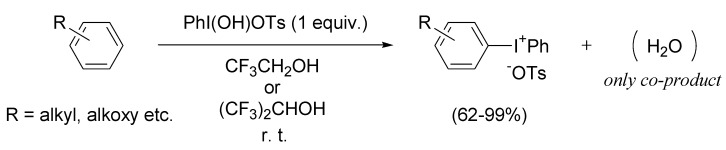
Synthesis of diaryliodonium salts using HTIB in fluoroalcohol solvents.

Recently, we found a remarkable rate-accelerating effect of the fluoroalcohol solvents for the direct dehydrative condensation of HTIB with various aromatic compounds, forming the corresponding diaryliodonium(III) salts as the products and water as waste (Scheme 3) [30]. We now report the spectroscopic study of the intermediates in the fluoroalcohol solvents to establish the SET oxidation ability of HTIB. Based on this study, our greener synthetic method of diaryliodonium salts was successfully applied to the various thiophenes that are sensitive to the SET oxidations [21,22,23,24].

## 2. Results and Discussion

### 2.1. Generation of aromatic cation radicals of phenyl ethers by HTIB

**Figure 2:**
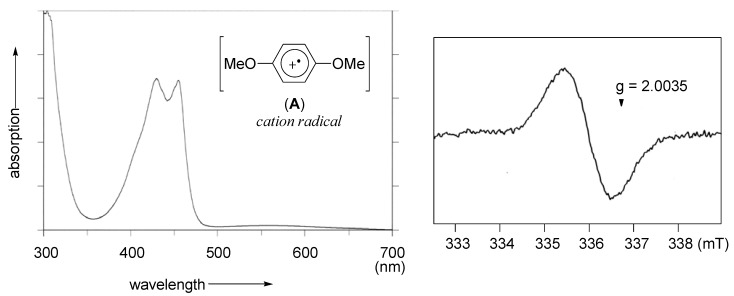
UV-VIS and ESR spectrums for the mixture of 1,4-dimethoxybenzene and HTIB in (CF_3_)_2_CHOH at room temperature.

We first examined the reaction of 1,4-dimethoxybenzene and HTIB in (CF_3_)_2_CHOH. The transparent solution of 1,4-dimethoxybenzene immediately changed to a green color with the addition of HTIB, and the disappearance of the starting material was confirmed by analytical TLC. We assumed that this observation implied the generation of a cation radical intermediate [18], and thus attempted to detect the intermediate by UV-VIS spectroscopic measurement. The spectrum showed a typical strong absorption band in the visible region between 400 and 500 nm, which shows good agreement with the reported value of the aromatic cation radical (**A**) (Figure 2, left) [18]. Furthermore, the radical species was detected by the ESR spectroscopic measurement, supporting the SET oxidation processes (Figure 2, right). Under the conditions, various diaryliodonium(III) salts were obtained from phenyl ethers and HTIB [30].

### 2.2. Application to the synthesis of thienyliodonium(III) salts

We next intended to apply this SET oxidation process for the synthesis of various thienyliodonium salts. The iodonium salts having thienyl moieties show a wide range of applicability as photoacid generator (PAG) for cationic polymerization [31], active bacteriocide [32,33], organic non-linear optic material [34], precursor of fluorinated aromatic compounds [35,36], *etc*. Very recently, we developed a novel metal-free oxidative cross-biaryl-coupling method of heteroaromatic compounds, in which thienyliodonium salts were involved as the key intermediates [37]. For their synthesis, the direct syntheses of the salts from thiophenes and activated iodine(III) reagents or HTIB were typically achieved for a limited number of substrates in moderate yields [38,39,40,41,42]. 

**Figure 3:**
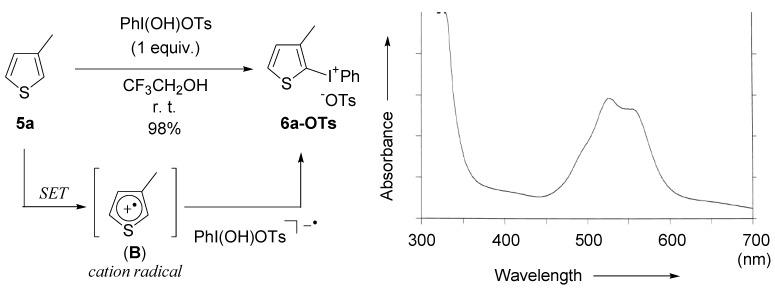
UV-VIS spectrum of the reaction of 3-methylthiophene (**5a**) using HTIB in CF_3_CH_2_OH. (The reaction was performed using an equimolar amount of thiophene **5a** and HTIB in CF_3_CH_2_OH (0.20M) at room temperature.)

Therefore, we examined the reaction of 3-methylthiophene (**5a**) and HTIB in CF_3_CH_2_OH. The reaction completely finished in a short time at room temperature and the thienyliodonium salt **6a **was isolated in an excellent yield after the purification by precipitation, while bithiophene was not found in this system because of the absence of Lewis acids that cause biaryl coupling reactions, such as BF_3_·Et_2_O and TMSOTf [22]. Other solvents, such as CH_2_Cl_2_, MeOH, and CH_3_CN, provided poorer results under the same reaction conditions (16%, 46%, and 23% yields, respectively). In the UV/VIS measurement, the spectrum of the reaction mixture between 500 and 600 nm of wavelength is in accordance with the absorption band of cation radical species (**B**) from the thiophene (Figure 3) [43].

This activating protocol of HTIB provided a facile access to a wide range of thienyliodonium salts **6** as shown in Table 1. The condensation preferentially occurred at the α-position of the sulfur atoms in the thiophene rings **5a-d** that are sensitive to the SET oxidations [21,22], among which the 2-positions of the cation radicals are known to be more reactive than the 5-positions (Entries 1–4) [23,24]. 3-Methoxythiophene (**5e**) was smoothly converted to the corresponding thienyliodonium salt **6e** in excellent yield (Entry 5). This is surprising because oxidative transformation of highly electron-rich alkoxythiophenes is limited owing to difficulty in suppressing uncontrollable polymerizations [44]. Even the substrates **5f**-**h** containing halogen, aryl, and ester groups, were converted to products in good yields with complete regioselectivity (Entries 6–8). Furthermore, 3,4-ethylenedioxythiophene (EDOT) (**5j**), which attracted attention due to its unique electronic characteristics, was readily functionalized (Entries 10 and 11), and the obtained diaryliodonium salt **6j** has significant prospects as an organic non-linear optic material [34]. For the α-disubstituted thiophene **5l**, on the other hand, the β-thienyl iodonium salt **6l **was obtained (Entry 12). A unique selectivity was observed for the organosilicon compound **5m**. For the competitive reaction of the C-H and C-Si bonds in 3-trimethylsilylthiophene (**5m**), an *ipso*-substitution product at the silicon-bound carbon as a result of electrophilic substitution *via* the Wheland type of σ-complex [38,39,40] was not obtained, but instead produced a dehydrative condensation product **6m** (Entry 13). No *ipso*-substitution product was obtained for the α-silyl substituted thiophene **5n**, while the condensation product **6n** and a small amount of the regioisomer were obtained (Entry 14). 

**Scheme 4:**
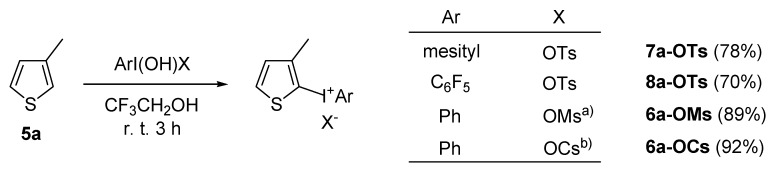
Use of several Koser’s-type reagents. (Reactions were performed using an equimolar amount of 3-methylthiophene (**5a**) and ArI(OH)X in CF_3_CH_2_OH (0.20M) at room temperature. a) Ms = methanesulfonyl. b) Cs = (+)-10-camphorsulfonyl.)

A significant number of Koser’s-type reagents are now readily available [45,46,47,48], and variation of the reagents in the reactions could expand the structural diversity of the obtained product. Selected examples are shown in Scheme 4. Thienyliodonium salts having different aryl rings and counterions, X, were obtained in excellent yields from a single thiophene substrate **5a **and the reagents, ArI(OH)X. As mentioned above, the present direct and waste-free approach based on the cation radical strategy has a broad range of versatility for the synthesis of various heteroaromatic diaryliodonium salts with a unique selectivity not seen in other synthetic methods.

**Table 1:** Scope of thiophene substrates.^a^

Entry	Thiophene	Thienyliodonium salt	Yield (%)^b^
	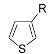	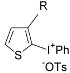	
1	R = Me ( **5a**)	**6a-OTs**	98
2	R = Hexyl ( **5b**)	**6b-OTs**	84
3	R = *i*-Buyl (**5c**)	**6c-OTs**	93
4	R = *c*-Hex (**5d**)	**6d-OTs**	74
5	R = OMe ( **5e**)	**6e-OTs**	89
6	R = Br ( **5f)**	**6f-OTs**	95
7	R = Ph ( **5g**)	**6g-OTs**	88
8	R = 4-MeO_2_CC_6_H_4_ ( **5h**)	**6h-OTs**	98
9	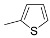	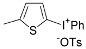	
	( **5i**)	**(6i-OTs)**	93
10	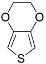	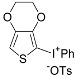	94
	( **5j**)	**(6j-OTs)**	
11	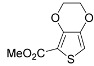	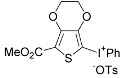	88
	( **5k**)	**(6k-OTs)**	
12	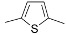	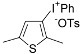	62
	( **5l**)	**(6l-OTs)**	
13	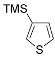	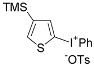	71
	( **5m**)	**(6m-OTs)**	
14	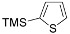	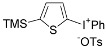	91^c^
	( **5n**)	**(6n-OTs)**	

^a^ Reactions were performed using equimolar amounts of the thiophenes **5** and PhI(OH)OTs in CF_3_CH_2_OH (0.20M) for 3 h at room temperature; ^b^ Isolated yield of pure products after precipitation; ^c^ Small amount of regioisomer was included.

## 3. Conclusions

In conclusion, we performed a detailed study of the enhanced reactivity of [hydroxy(tosyloxy)iodo] benzene (HTIB) in fluoroalcohol media, resulting in the first establishment of the SET oxidation ability of HTIB by successive spectroscopic detection of the aromatic cation radicals. The use of the activation protocol of HTIB by fluoroalcohol solvents led to the efficient synthesis of diaryliodonium salts from electron-rich heteroaromatic compounds represented by the thiophenes. This facile and clean preparative method of diaryliodonium salts would expand the possibility of heteroaromatic compounds as a unique aryl source in the field of organic synthesis.

## 4. Experimental

Melting points (mp) are uncorrected. The ^1^H-NMR and ^13^C-NMR spectra were recorded by a JEOL JMN-300 or JMN-400 spectrometers operating at 300 or 400 MHz (75.3 or 100.53 MHz for ^13^C-NMR) in CDCl_3_ or CD_3_OD at 25 °C with tetramethylsilane as the internal standard. The data are reported as follows: chemical shift in part par million (δ), multiplicity (s = singlet, d = doublet, t = triplet, q = quartet, br = broad singlet, m = multiplet), integration, and coupling constant (Hz). The infrared spectra (IR) were obtained using a Hitachi 270-50 spectrometer. The mass spectra were obtained using a Shimadzu GCMS-QP 5000 instrument with ionization voltages of 70 eV. The high resolution mass spectra and elemental analysis were performed by the Elemental Analysis Section of Osaka University. ESR spectra were taken on JEOL JES-RE 1X spectrometer. PhI(OH)OTs (HTIB, Koser’s reagent) is a commercially available compound and was used as received. Thiophene **5h** was prepared by Suzuki coupling of 3-thiophene-boronic acid with methyl 4-iodobenzoate [49]. Solvents and all other starting materials were obtained from commercial suppliers and used without further purification.

### 4.1. Measurement of UV-VIS absorption spectrum

To a stirred solution of 1,4-dimethoxybenzene (1.4 mg, 1.0 × 10^-2^ mmol) in (CF_3_)_2_CHOH (5 mL) was added HTIB (3.9 mg, 1.0 × 10^-2^ mmol) in one portion at room temperature under air. The UV-VIS absorption spectrum of the reaction mixture was measured on Shimadzu 2200 UV-VIS spectrometer.

### 4.2. Measurement of electron spin resonance spectrum

Under air at room temperature, to a stirred solution of **6a** (2.0 mg, 2.0 × 10^-2^ mmol) in CF_3_CH_2_OH (2 mL) was added HTIB (7.8 mg, 2.0 × 10^-2^ mmol) in one portion, and the mixture was inserted into the ESR cavity. The spectrum was recorded at room temperature on a JEOL JES-RE 1X spectrometer. Instrument conditions were as follows: magnetic field, 336 ± 5 mT; modulation frequency, 0.05 mT; modulation amplitude, 100 kHz; output power, 1.4 mW; time constant, 0.1 sec; sweep time, 1 min/10 mT; amplitude, 32.

### 4.3. General procedure for the preparation of diaryliodonium salts

To a stirred solution of **5a** (98 mg, 1 mmol) in CF_3_CH_2_OH (5 mL), [hydroxyl(tosyloxy)iodo] benzene (392 mg, 1 mmol) was added in one portion at room temperature under air, and it was stirred for 3 h. MeOH was then added to the reaction mixture when the solvents were removed under vacuum. The resulting oily crude product **6a-OTs** was precipitated by adding Et_2_O with stirring. The precipitate was collected and dried in *vacuo* to give **6a-OTs** (396 mg, 84%) as a slightly gray powder.

*(3-Methyl-2-thienyl)(phenyl)iodonium tosylate* (**6a-OTs**) [30,39]. A slightly gray solid, mp 165 °C. IR (KBr) cm^-1^: 3051, 1575, 1469, 1440, 1377, 1191, 1132, 1045, 1014, 991, 815, 746, 680. ^1^H-NMR (300 MHz, CD_3_OD) δ 2.33 (s, 3H), 2.49 (s, 3H), 7.03 (d, 1H, *J* = 5.1 Hz), 7.19 (d, 2H, *J* = 7.2 Hz), 7.46-7.49 (m, 2H), 7.59-7.67(m, 3H), 7.83 (d, 1H, *J* = 5.1 Hz,), 8.05 (d, 2H, *J* = 7.8 Hz) ppm. ^13^C-NMR (75.5 MHz, CD_3_OD) δ 17.5, 21.3, 98.4, 118.4, 126.9, 129.8, 131.0, 133.0, 133.1, 133.4, 135.4, 137.7, 141.6, 150.0 ppm. 

*(3-Hexyl-2-thienyl)(phenyl)iodonium tosylate* (**6b-OTs**). A colorless solid, mp 145-146 °C. IR (KBr) cm^-1^: 3047, 2931, 1525, 1454, 1437, 1381, 1192, 1132, 1045, 1014, 910, 815, 732, 696. ^1^H-NMR (300 MHz, CD_3_OD) δ 0.84 (t, 3H, *J* = 5,4 Hz), 1.16-1.24 (m, 6H), 1.42-1.46 (m, 2H), 2.32 (s, 3H), 2.72 (t, 2H, *J* = 6.3 Hz), 6.94 (d, 1H, *J* = 4.2 Hz), 7.07 (d, 2H, *J* = 8.4 Hz), 7.33 (t, 2H, *J* = 7.8 Hz), 7.47 (t, 1H, *J* = 7.8 Hz), 7.56 (d, 2H, *J* = 7.8 Hz), 7.63 (d, 1H, *J* = 4.2 Hz), 7.87 (d, 2H, *J* = 8.4 Hz) ppm. ^13^C-NMR (75.5 MHz, CD_3_OD) δ 14.0, 21.3, 22.4, 28.9, 30.3, 31.5, 31.9, 97.5, 118.6, 126.0, 128.5, 128.8, 131.3, 131.6, 133.3, 136.1, 139.6, 142.2, 153.4 ppm. HRMS (FAB): Calcd for C_13_H_12_IO [M-OTs]^+^: 371.0330, found 371.0339. 

*(3-Isobutyl-2-thienyl)(phenyl)iodonium tosylate* (**6c-OTs**). Colorless amorphous. IR (KBr) cm^-1^: 3045, 2951, 1464, 1438, 1384, 1190, 1132, 1045, 912, 815, 731, 696. ^1^H-NMR (400 MHz, CDCl_3_) δ 0.84 (d, 6H, *J* = 4.8 Hz), 1.86 (m, 1H), 2.33 (s, 3H), 2.62 (d, 2H, *J* = 4.8 Hz), 6.96 (d, 1H, *J* = 5.6 Hz), 7.11 (d, 2H, *J* = 8.4 Hz), 7.36 (t, 2H, *J* = 8.0 Hz), 7.49 (t, 1H, *J* = 8.0 Hz), 7.63 (d, 2H, *J* = 8.0 Hz), 7.68 (1H, *J* = 5.6 Hz), 7.89 (d, 2H, *J* = 8.4 Hz) ppm. ^13^C-NMR (100.53 MHz, CDCl_3_) δ 21.3, 22.2, 29.8, 40.6, 97.5, 118.6, 126.0, 128.7, 129.4, 131.6, 131.8, 133.2, 136.4, 140.0, 141.8, 152.8 ppm. HRMS (FAB) Calcd for C_14_H_16_IS [M-OTs]^+^ 343.0017, found 343.0015.

*(3-Cyclohexyl-2-thienyl)(phenyl)iodonium tosylate* (**6d-OTs**). A colorless solid, mp 128–130 °C (ether). IR (KBr) cm^-1^: 3053, 2926, 2850, 1564, 1469, 1440, 1190, 1132, 1045, 991, 914, 815, 732. ^1^H-NMR (400 MHz, CDCl_3_) δ 1.22-1.77 (m, 10H), 2.33 (s, 3H), 2.76 (m, 1H), 6.97 (d, 1H, *J* = 5.6 Hz), 7.09 (d, 2H, *J* = 8.0 Hz), 7.35 (t, 2H, *J* = 8.0 Hz), 7.48 (t, 1H, *J* = 8.0 Hz), 7.61 (d, 2H, *J* = 8.0 Hz), 7.68 (d, 1H, *J* = 5.6 Hz), 7.88 (d, 2H, *J* = 8.0 Hz) ppm. ^13^C-NMR (100.53 MHz, CDCl_3_) δ 21.3, 25.6, 26.1, 34.1, 42.0, 96.4, 118.9, 126.0, 127.0, 128.7, 131.5, 131.8, 133.3, 136.6, 139.9, 141.9, 158.4 ppm. HRMS (FAB): Calcd for C_16_H_18_IS [M-OTs]^+^: 369.0174, found 369.0165

*(3-Methoxy-2-thienyl)(phenyl)iodonium tosylate* (**6e-OTs**). A blue solid, mp 49 °C. IR (KBr) cm^-1^: 3014, 1554, 1471, 1379, 1217, 1132, 1070, 1043, 1014, 771, 694. ^1^H-NMR (300 MHz, CD_3_OD) δ 2.35 (s, 3H), 4.02 (s, 3H), 7.08 (d, 1H, *J* = 6.0 Hz), 7.21 (d, 2H, *J* = 7.8 Hz), 7.46-7.51 (m, 2H), 7.61-7.69 (m, 3H), 7.97 (d, 1H, *J* = 6.0 Hz), 8.03 (d, 2H, *J* = 7.8 Hz) ppm. ^13^C-NMR (75.5 MHz, CD_3_OD) δ 21.3, 60.4, 77.4, 116.5, 118.7, 126.9, 129.8, 133.0, 133.4, 135.5, 138.7, 141.6, 143.6, 165.1 ppm. HRMS (FAB): Calcd for C_11_H_10_IOS [M-OTs]^+^: 316.9497, found 316.9504.

*(3-Bromo-2-thienyl)(phenyl)iodonium tosylate* (**6f-OTs**). A colorless solid, mp 49 °C. IR (KBr) cm^-1^: 3045, 1562, 1469, 1438, 1373, 1330, 1191, 1130, 1043, 1014, 860, 815, 740, 692. ^1^H-NMR (300 MHz, CD_3_OD) δ 2.35 (s, 3H), 7.19-7.24 (m, 3H), 7.53 (t, 2H, *J* = 7.8 Hz), 7.67 (d, 3H, *J* = 7.8 Hz), 7.98 (d, 1H, *J* = 5.7 Hz), 8.16 (d, 2H, *J* = 7.8 Hz) ppm. ^13^C-NMR (75.5 MHz, CD_3_OD) δ 21.3, 119.2, 125.6, 126.9, 129.8, 132.1, 133.2, 133.9, 135.9, 139.8, 141.4, 141.7, 143.5 ppm. HRMS (FAB): Calcd for C_10_H_7_BrIS [M-OTs]^+^: 364.8497, found 364.8501.

*(3-Phenyl-2-thienyl)(phenyl)iodonium tosylate* (**6g-OTs**) [30]. A colorless solid, mp 122–123 °C. IR (KBr) cm^-1^: 3053, 1485, 1469, 1440, 1265, 1197, 1132, 1045, 1014, 991, 815, 748, 696. ^1^H-NMR (300 MHz, CD_3_OD): δ 2.31 (s, 3H), 7.12-7.24 (m, 3H), 7.31 (t, 2H,* J* = 7.5 Hz), 7.37-7.47 (m, 2H), 7.48-7.62 (m, 6H), 7.65 (d, 2H,* J* = 7.8 Hz), 8.00 (d, 1H,* J* = 5.1 Hz) ppm. ^13^C-NMR (75.5 MHz, CD_3_OD) δ 21.3, 98.7, 118.7, 126.9, 129.8, 130.2, 130.4, 130.5, 132.7, 133.3, 135.2, 135.6, 138.5, 141.6, 143.3, 153.2 ppm. HRMS (FAB): Calcd for C_16_H_12_IS [M-OTs]^+^: 362.9704, found 362.9702.

*[3-(4-Methoxycarbonyl)phenyl-2-thienyl](phenyl)iodonium tosylate* (**6h-OTs**). A slightly yellow solid, mp 126–128 °C (ether). IR (KBr) cm^-1^: 3051, 1722, 1608, 1438, 1280, 1188, 1116, 1045, 1016, 912, 734. ^1^H-NMR (400 MHz, CDCl_3_) δ 2.30 (s, 3H), 3.98 (s, 3H), 7.03 (d, 2H, *J* = 7.6 Hz), 7.09 (d, 1H, *J* = 5.6 Hz), 7.18 (t, 2H, *J* = 7.6 Hz), 7.39 (t, 1H, *J* = 7.6 Hz), 7.49 (m, 6H), 7.72 (d, 1H, *J* = 5.6 Hz), 8.08 (d, 2H, *J* = 7.6 Hz) ppm. ^13^C-NMR (100.53 MHz, CDCl_3_) δ 21.3, 52.3, 99.7, 118.9, 120.1, 126.0, 128.6, 129.2, 130.2, 130.5, 131.3, 131.4, 133.9, 136.3, 138.7, 140.1, 141.4, 150.4, 166.5 ppm. HRMS (FAB): Calcd for C_18_H_14_IO_2_S [M-OTs]^+^: 420.9759, found 420.9778.

*(5-Methyl-2-thienyl)(phenyl)iodonium tosylate* (**6i-OTs**). A brown solid, mp 89–92 °C (ether). IR (KBr) cm^-1^: 3053, 1440, 1193, 1128, 1039, 1012, 817, 792, 738, 680 ^1^H-NMR (400 MHz, CDCl_3_) δ 2.30 (s, 3H), 2.51 (s, 3H), 6.65 (d, 1H, *J* = 3.6 Hz), 7.05 (d, 2H, *J* = 8.4 Hz), 7.30 (t, 2H, *J* = 8.0 Hz), 7.46 (t, 1H, *J* = 8.0 Hz), 7.54 (d, 2H, *J* = 8.4 Hz), 7.64 (d, 1H, *J* = 3.6 Hz), 7.93 (d, 2H, *J* = 8.0 Hz) ppm. ^13^C- NMR (100.53 MHz, CDCl_3_) δ 15.4, 21.3, 93.5, 118.3, 126.0, 128.1, 128.7, 131.5, 131.6, 134.0, 140.3, 140.9, 141.7, 152.2 ppm. HRMS (FAB): Calcd for C_11_H_10_S [M-OTs]^+^: 300.9548, found 300.9548.

*(2,3-dihydro-thieno[3,4-b][1,4]dioxin-5-yl)(phenyl)iodonium tosylate* (**6j-OTs**). A blue solid, mp 48–49 °C. IR (KBr) cm^-1^: 2943, 1485, 1359, 1188, 1132, 1062, 1045, 1014, 750, 694. ^1^H-NMR (300 MHz, CDCl_3_) δ 2.33 (s, 3H), 4.18-4.22 (m, 2H), 4.27-4.32 (m, 2H), 6.77 (s, 1H), 7.09 (d, 2H, *J* = 8.4Hz), 7.35 (t, 2H, *J* = 7.8 Hz), 7.49 (t, 1H, *J* = 7.8 Hz), 7.59 (d, 2H, *J* = 7.8 Hz), 7.96 (d, 2H, *J* = 8.4 Hz) ppm. ^13^C-NMR (75.5 MHz, CD_3_OD) δ 21.3, 64.4, 65.6, 74.1, 111.4, 119.3, 126.0, 128.6, 131.4, 131.6, 133.7, 139.7, 141.3, 142.1, 148.0 ppm. HRMS (FAB): Calcd for C_12_H_10_IO_2_S [M-OTs]^+^: 344.9446, found 344.9450. 

*(7-Methoxycarbonyl-2,3-dihydro-thieno[3,4-b][1,4]dioxin-5-yl)(phenyl)iodonium tosylate* (**6k-OTs**). A colorless solid, mp 169 °C (ether). IR (KBr) cm^-1^: 3520, 3051, 2949, 1712, 1573, 1487, 1444, 1359, 1274, 1193, 1089, 912, 740. ^1^H-NMR (400 MHz, CDCl_3_) δ 2.31 (s, 3H), 3.83 (s, 3H), 4.28-4.32 (m, 4H), 7.02 (d, 2H, *J* = 7.6 Hz), 7.32 (t, 2H, *J* = 7.6 Hz), 7.45 (m, 3H), 7.96 (d, 2H, *J* = 7.6 Hz) ppm. ^13^C- NMR (100.53 MHz, CDCl_3_) δ 21.2, 52.2, 65.0, 65.1, 83.8, 99.9, 116.0, 118.6, 125.8, 128.4, 131.4, 134.5, 139.7, 141.9, 144.1, 146.4, 160.2 ppm. HRMS (FAB): Calcd for C_14_H_12_IO_4_S [M-OTs]^+^: 402.9501, found 402.9508.

*(2,5-Dimethyl-3-thienyl)(phenyl)iodonium tosylate* (**6l-OTs**). A colorless solid, mp 111–112 °C (ether). IR (KBr) cm^-1^: 3539, 3047, 2918, 1562, 1469, 1193, 1132, 1045, 912, 815, 740. ^1^H-NMR (400 MHz, CDCl_3_) δ 2.31 (s, 3H), 2.36 (s, 3H), 2.52 (s, 3H), 6.87 (s, 1H), 7.04 (d, 2H, *J* = 8.0 Hz), 7.31 (t, 2H, *J* = 7.6 Hz), 7.44 (t, 1H, *J* = 7.6 Hz), 7.50 (d, 2H, *J* = 8.0 Hz), 7.84 (d, 2H, *J* = 7.6 Hz) ppm. ^13^C-NMR (100.53 MHz, CDCl_3_) δ 15.2, 17.0, 21.2, 99.6, 115.9, 125.9, 128.4, 129.5, 131.0, 131.4, 133.9, 139.3, 141.2, 142.6, 145.5 ppm. HRMS (FAB): Calcd for C_12_H_12_IS [M-OTs]^+^: 314. 9704, found 314. 9703.

*Phenyl(4-trimethylsilyl-2-thienyl)iodonium tolsylate* (**6m-OTs**). A colorless solid, mp 121 °C. IR (KBr) cm^-1^: 3051, 2954, 1566, 1469, 1440, 1253, 1199, 1132, 1103, 1043, 1014, 991, 883, 842, 750, 680. ^1^H-NMR (300 MHz, CDCl_3_) δ 0.32 (s, 9H), 2.40 (s, 3H), 7.26 (d, 2H, *J* = 7.8 Hz), 7.54 (t, 2H, *J* = 7.5 Hz), 7.67-7.74 (m, 3H), 7.99 (s, 1H), 8.11 (s, 1H), 8.19 (d, 2H, *J* = 7.8 Hz) ppm. ^13^C-NMR (75.5 MHz, CD_3_OD) δ -0.59, 21.6, 119.1, 127.2, 130.1, 133.4, 133.9, 136.1, 136.4, 141.9, 143.8, 144.4, 146.5, 147.0 ppm. HRMS (FAB): Calcd for C_13_H_16_ISSi [M+H]^+^: 358.9781, found 358.9792.

*Phenyl(5-trimethylsilyl-2-thienyl)iodonium tolsylate* (**6n-OTs**, including a small amount of regioisomer). A colorless solid. IR (KBr) cm^-1^: 3055, 2955, 1494, 1469, 1440, 1392, 1195, 1120, 1045, 1008, 985, 842, 738, 680. ^1^H-NMR (300 MHz, CD_3_OD) δ 0.37 (s, 9H), 2.40 (s, 3H), 7.25 (d, 2H,*J* = 7.8 Hz), 7.34 (d, 1H, *J* = 3.3 Hz), 7.56 (t, 2H, *J* = 7.5 Hz), 7.69-7.74 (m, 3H), 8.04 (d, 1H, *J* = 3.3 Hz), 8.20 (d, 2H, *J* = 7.8 Hz) ppm. ^13^C-NMR (75.5 MHz, CD_3_OD) δ 0.43, 22.1, 103.5, 119.9, 127.8, 130.7, 134.0, 134.6, 136.7, 138.3, 142.5, 143.7, 144.4, 156.5 ppm.

*(3-Methyl-2-thienyl)(2,4,6-trimethylphenyl)iodonium tosylate* (**7a-OTs**). A colorless solid, mp 145 °C (ether). IR (KBr) cm^-1^: 3637, 3466, 3061, 2953, 1923, 1600, 1450, 1377, 1300, 1215, 1132, 1043, 815, 758. ^1^H-NMR (300 MHz, CDCl_3_) δ 2.27 (s, 3H), 2.30 (s, 3H), 2.47 (s, 3H), 2.67 (s, 6H), 6.84 (d, 1H, *J* = 5.4 Hz), 6.93 (s, 2H), 7.00 (d, 2H, *J* = 7.8 Hz), 7.40 (d, 2H, *J* = 7.8 Hz), 7.48 (d, 1H, *J* = 5.4 Hz) ppm. ^13^C-NMR (75.3 MHz, CDCl_3_) δ 17.7, 20.9, 21.2, 27.1, 96.9, 125.8, 126.4, 128.3, 129.6, 129.7, 134.3, 139.2, 141.2, 142.3, 143.1, 147.2 ppm. HRMS (FAB): Calcd for C_14_H_16_IS [M-OTs]^+^: 343.0017, found 343.0026.

*(3-Methyl-2-thienyl)(pentafluorophenyl)iodonium tosylate* (**8a-OTs**). A colorless solid, mp 140–141 °C. (ether). IR (KBr) cm^-1^: 3091, 1633, 1487, 1390, 1195, 1078, 974, 908, 815, 756, 696. ^1^H-NMR (300 MHz, CDCl_3_) δ 2.35 (s, 3H), 2.49 (s, 3H), 6.81 (d, 1H, *J* = 5.4 Hz), 7.08 (d, 2H, *J* = 7.8 Hz), 7.37 (d, 2H, *J* = 7.8 Hz), 7.46 (d, 1H, *J* = 5.4 Hz) ppm. ^13^C-NMR (75.3 MHz, CDCl_3_) δ 17.5, 21.2, 93.7, 100.0, 125.7, 128.6, 129.4, 135.5, 138.0, 140.4, 140.6, 144.3, 146.8, 149.1 ppm. HRMS (FAB): Calcd for C_11_H_5_ F_5_IS [M-OTs]^+^: 390. 9077, found 390.9095.

*(3-Methyl-2-thienyl)(phenyl)iodonium methanesulfonate* (**6a-OMs**). A colorless solid, mp 144 °C (ether). IR (KBr) cm^-1^: 3047, 1562, 1525, 1469, 1440, 1377, 1327, 1222, 1053, 991, 912, 825, 785, 742. ^1^H-NMR (300 MHz, CDCl_3_) δ 2.51 (s, 3H), 2.52 (s, 3H), 6.97 (d, 1H, *J* = 5.1 Hz), 7.40 (t, 2H, *J* = 7.5 Hz), 7.51 (t, 1H, *J* = 7.5 Hz), 7.62 (d, 1H, *J* = 5.1 Hz), 7.94 (d, 2H, *J* = 7.5 Hz) ppm. ^13^C-NMR (75.3 MHz, CDCl_3_) δ 17.5, 39.0, 98.3, 1178.0, 129.8, 131.3, 131.5, 133.6, 135.7, 148.4 ppm. HRMS (FAB): Calcd for C_11_H_10_IS [M-OMs]^+^: 300.9548, found 300.9550.

*(3-Methyl-2-thienyl)(pheny)iodonium (+)-10-camphorsulfonate* (**6a-OCs**). A colorless solid, mp 145 °C (ether). IR (KBr) cm^-1^: 3458, 2956, 1732, 1562, 1469, 1373, 1192, 1051, 918, 732. ^1^H-NMR (400 MHz, CDCl_3_) δ 0.77 (s, 3 H), 1.01 (s, 3H), 1.26-1.32 (m, 1H), 1.52-1.59 (m, 1H), 1.83 (d, 1H, *J* = 18.4 Hz), 1.89-2.00 (m, 2H), 2.25-2.32 (m, 1H), 2.51 (s, 3H), 2.54-2.62 (m, 1H), 2.68 (d, 1H, *J* = 14.8 Hz), 3.21 (d, 1H, *J* = 14.8 Hz), 6.98 (d, 1H, *J* = 5.2 Hz), 7.40 (t, 2H, *J* = 8.0 Hz), 7.50 (t, 1H, *J* = 8.0 Hz), 7.63 (d, 1H, *J* = 5.2 Hz), 7.96 (d, 2H, *J* = 8.0 Hz) ppm. ^13^C-NMR (100.53 MHz, CDCl_3_) δ 17.5, 19.7, 19.9, 24.3, 27.0, 42.6, 42.8, 47.2, 47.7, 58.5, 98.3, 118.6, 130.0, 131.3, 131.6, 133.6, 135.7, 148.7, 217.0 ppm. HRMS (FAB): Calcd for C_11_H_10_IS [M-OCs]^+^: 300.9548, found 300.9570.
